# Effects of Angiogenic Factors on the Epithelial-to-Mesenchymal Transition and Their Impact on the Onset and Progression of Oral Squamous Cell Carcinoma: An Overview

**DOI:** 10.3390/cells13151294

**Published:** 2024-07-31

**Authors:** Silvia Pomella, Ombretta Melaiu, Maria Dri, Mirko Martelli, Marco Gargari, Giovanni Barillari

**Affiliations:** 1 Department of Clinical Sciences and Translational Medicine, University of Rome Tor Vergata, Via Montpellier, 00133 Rome, Italy; silvia.pomella@uniroma2.it (S.P.); ombretta.melaiu@uniroma2.it (O.M.); mirko.marte@libero.it (M.M.); marco.gargari@gmail.com (M.G.); 2 Department of Surgical Sciences, University of Rome Tor Vergata, 00133 Rome, Italy; mariampdri6@gmail.com

**Keywords:** VEGF, FGF-2, ANG-2, AKT, MAPK, EMT, OPMD, OSCC, angiogenesis, metastasis

## Abstract

High levels of vascular endothelial growth factor (VEGF), fibroblast growth factor (FGF)-2 and angiopoietin (ANG)-2 are found in tissues from oral squamous cell carcinoma (OSCC) and oral potentially malignant disorders (OPMDs). As might be expected, VEGF, FGF-2, and ANG-2 overexpression parallels the development of new blood and lymphatic vessels that nourish the growing OPMDs or OSCCs and provide the latter with metastatic routes. Notably, VEGF, FGF-2, and ANG-2 are also linked to the epithelial-to-mesenchymal transition (EMT), a trans-differentiation process that respectively promotes or exasperates the invasiveness of normal and neoplastic oral epithelial cells. Here, we have summarized published work regarding the impact that the interplay among VEGF, FGF-2, ANG-2, vessel generation, and EMT has on oral carcinogenesis. Results from the reviewed studies indicate that VEGF, FGF-2, and ANG-2 spark either protein kinase B (AKT) or mitogen-activated protein kinases (MAPK), two signaling pathways that can promote both EMT and new vessels’ formation in OPMDs and OSCCs. Since EMT and vessel generation are key to the onset and progression of OSCC, as well as to its radio- and chemo-resistance, these data encourage including AKT or MAPK inhibitors and/or antiangiogenic drugs in the treatment of this malignancy.

## 1. Introduction

Squamous cell carcinoma (SCC) is an aggressive tumor that, together with other head and neck neoplasms, ranks sixth among the most common human malignancies worldwide, representing over 90% of those arising in the oral cavity (oral SCC, OSCC) [1].

In most cases, the onset of an OSCC is preceded by that of dysplastic–hyperplastic lesions, termed oral potentially malignant disorders (OPMDs) [1]. Among them, non-homogeneous leukoplakia, erythroplakia, proliferative verrucous leukoplakia, and submucous fibrosis have a high risk of transforming into OSCC [2].

The development of an OPMD and its eventual evolution into an OSCC, as well as OSCC’s clinical progression, are preceded and accompanied by the chronic inflammation of oral mucosa that results from prolonged exposure to mechanical traumas (e.g., broken or badly positioned teeth), chemical substances (e.g., tobacco and/or ethyl alcohol), or infectious agents (e.g., bacteria causing periodontitis) [1,3,4]. Such a persistent inflammation leads to the epithelial-to-mesenchymal transition (EMT) of oral epithelial cells [5,6,7].

EMT is a cellular trans-differentiation process impelling epithelial cells to gradually lose their static phenotype (that is firmly attached to sister cells and to the basement membrane and oriented in an apical-basal polarity), and to acquire the invasive and migratory capabilities that are peculiar to mesenchymal cells [8,9,10,11,12].

When it is transient and reversible, EMT plays an important role in tissue damage repair [11]. On the other hand, long-lasting, intense EMT is associated with inflammatory, degenerative, or neoplastic diseases [6,8,9,10,12,13,14].

Epithelial cells that undergo EMT lose epithelial (E)-cadherin, a receptor that mediates homotypic adhesion between epithelial cells, and replace it with de novo synthesizing neuronal (N)-cadherin, an intercellular adhesion molecule expressed by mesenchymal cells: because of the E-cadherin loss, the adhesiveness between epithelial cells decreases [6,7,8,9,10]. Such a change in the expression of cadherins, which is known as the “cadherin switch”, characterizes OSCC tissues; its intensity is directly proportional to the ability of the tumor to metastasize and/or relapse after surgical removal [6].

Moreover, during the EMT process, the expression of vimentin (a component of mesenchymal cell cytoskeleton) is induced, which contributes to conferring mobility to the otherwise static epithelial cells [6,7,8,9,10].

Another feature of the EMT phenotype is the synthesis of the matrix metalloproteases (MMPs) and/or the urokinase plasminogen activator (uPA): these enzymes effectively degrade the molecular constituents of the extracellular matrix (ECM) and basement membranes, hence endowing the transdifferentiated epithelial cells with invasive capabilities [3,6,7,8,9,10,15,16].

In addition to EMT, the development and progression of OSCC are also accompanied by the neoformation of blood and lymphatic vessels [17,18,19,20]. As with many other tumor types, in OSCC the new blood vessels are formed mainly through angiogenesis [21], a multistep process in which endothelial cells lining the endoluminal face of the capillaries and venules degrade the vascular basement membrane and migrate into the perivascular space to form solid cords: the latter will then cavitate to allow the blood to flow into them [22]. The formation of new lymphatic vessels (lymphangiogenesis) occurs in ways that are like those of angiogenesis [22].

Angiogenesis and lymphangiogenesis are promoted by a variety of molecules which, taken together, are named angiogenic factors [22]. Among them, those belonging to the vascular endothelial growth factor (VEGF) family are particularly effective [23]. Specifically, VEGF-A is believed to be the most important mediator of angiogenesis, while VEGF-C is mainly involved in lymphangiogenesis [23,24]. Another family of cytokines that can promote all the events underlying angiogenesis is that of the fibroblast growth factors (FGFs): amidst them, FGF-2, also termed basic FGF, takes on particular importance in tumor angiogenesis [25]. Another effective angiogenic factor is angiopoietin-2 (ANG-2), which starts angiogenesis by destabilizing the pre-existing vessels [26,27].

As seen for EMT, angiogenesis and lymphangiogenesis are also pivotal to OSCC progression and metastasis. Through angiogenesis, in fact, the proliferating OPMDs or OSCCs are equipped with new vessels that add to the original ones to satisfy, at least in part, the increased need for oxygen and nutrients [24,28,29]. Consistently, the number of blood vessels is greater in a growing OPMD compared to healthy oral mucosa, and it further increases in an enlarging OSCC [30]. On its part, lymphangiogenesis provides OSCC cells with additional routes to metastasize [24,29]. In accordance, the intensity of lymphangiogenesis is a reliable indicator of metastasis risk and poor prognosis for OSCC patients [31]. Importantly, in OSCC tissues, tumor cells that have undergone EMT localize in the areas of lymphangiogenesis, from where they will metastasize [32].

Of utmost interest, EMT, angiogenesis, and lymphangiogenesis are simultaneously triggered during oral carcinogenesis [17,21,30,32,33,34,35,36]. In fact, in OPMDs and OSCCs, the number of newly formed vessels rises in parallel with the increase in the expression of EMT markers [30].

Based on these data, here, we have summarized and discussed the results from studies concerning the role that the angiogenic VEGF, FGF-2, and ANG-2 have in the EMT underlying the development, progression, and metastasizing of OSCC. Data were searched for in the PubMed Central electronic database of the National Library of Medicine (National Institutes of Health, Bethesda, Maryland, United States of America). The search was carried out from June 2023 to July 2024. There was no time restriction on the studies included, and the final data consisted of studies published from 1996 to 2024. In total, 161 articles were selected for full-text screening, and 135 of them were included in the final study. The other articles discussed and cited in this review served to complete the description of the topics considered herein.

## 2. Molecular Mechanisms Underlying EMT

**Figure 1:**
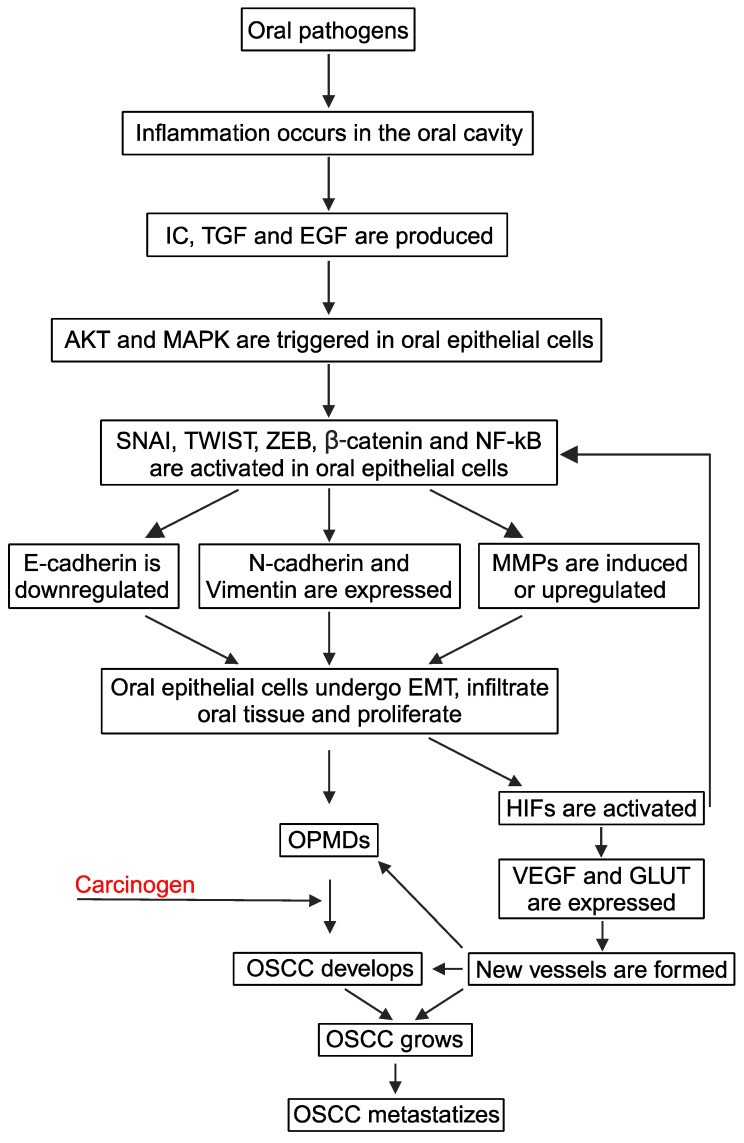
Molecular mechanisms leading to EMT in oral mucosa. Arrows symbolize directions of connections. Abbreviations: AKT, protein kinase B; E-cadherin, epithelial-cadherin; EGF, epidermal growth factor; EMT, epithelial-to-mesenchymal transition; GLUT, glucose transporter protein; HIF, hypoxia-inducible factor; IC, inflammatory cytokines; MAPK, mitogen-activated protein kinase; MMPs, matrix metalloproteinases; N-cadherin, neuronal-cadherin; NF-κB, nuclear factor kappa B; OPMDs, oral potentially malignant disorders; OSCC, oral squamous cell carcinoma; SNAI, zinc finger snail homolog; TGF, transforming growth factor; TWIST, basic helix–loop–helix twist homolog; VEGF, vascular endothelial growth factor; ZEB, zinc finger E-box-binding homeobox. Created with BioRender.com.

During chronic oral mucositis of mechanical, chemical, or microbial origin, leukocytes and macrophages are recruited in the damaged mucosa, where they produce cytokines such as interleukin-1, -6, and -8, tumor necrosis factors, transforming growth factor (TGF)-β1, and epidermal growth factor (EGF) [3] (Figure 1).

As for other inflamed epithelia, also in oral mucosa, the above-mentioned inflammatory mediators or growth factors turn on transcription factors such as the zinc finger snail homolog (SNAI), the basic helix–loop–helix twist homolog (TWIST), and the zinc finger E-box-binding homeobox (ZEB) in epithelial cells, which then acquire the EMT phenotype [3,37,38,39] (Figure 1). Specifically, SNAI, TWIST, or ZEB induces the expression of mesenchymal N-cadherin or vimentin and represses the synthesis of E-cadherin and other epithelial molecules [3,37,38,39]. At the same time, SNAI, TWIST, and ZEB promote MMPs expression [3,37,38,39]. In this context, one should consider that the EMT process is slow and reversible [40]: this explains why cells with epithelial or mesenchymal characteristics are concomitantly present in a chronically inflamed oral mucosa together with cells that show intermediate phenotypes between the decidedly epithelial and the predominantly mesenchymal one [41].

In addition to inducing EMT, inflammatory cytokines stimulate the growth of the trans-differentiated oral epithelial cells both directly and by amplifying the effects of growth factors [4].

While it is commonly believed that the EMT phenotype is more related to cell migration than to cell proliferation [8], it has recently been shown that EMT boosts the proliferation of epithelial cells cultured in 3D [42]. Another study indicates that, upon their exposure to TGF, epithelial cells undergo EMT and, at the same time, display increased viability and an increased growth rate [43]. Moreover, the inhibition of EMT in lung carcinoma cells is followed by a decrease in both their proliferation and invasiveness [44].

Overall, these events explain why dysplastic/hyperplastic OPMDs often originate from a persistent inflammation of the oral cavity [3,4,45].

In this context, environmental or infectious mutagens can promote the malignant transformation of the trans-differentiated and/or proliferating oral epithelial cells, leading to the onset of an OSCC: thus, EMT is related to the OPMD transformation into OSCC [4,45,46] (Figure 1).

As for the normal epithelial cells that line an inflamed oral cavity, and as for the dysplastic cells constituting OPMDs, the transformed OSSC cells will also be exposed for a prolonged time to cytokines and growth factors produced by infiltrating leukocytes and macrophages, by fibroblasts, and by the activated oral epithelial cells themselves: consequently, also OSCC cells undergo EMT; this exacerbates their invasive capabilities [3,4]. Similar to what happens with normal epithelial cells lining a chronically inflamed oral mucosa, OSCC cells can also display a variety of phenotypes ranging from fully epithelial to markedly mesenchymal ones [47,48]. Actually, in OSCC tissues, most OSCC cells display an intermediate phenotype since they express both epithelial and mesenchymal markers [47].

In a wide variety of epithelial cells, those lining the oral cavity included, the activation of EMT-inducing transcription factors is triggered by two intracellular signaling pathways, namely the phosphoinositide 3-kinase (PI3K)/protein kinase B (AKT) and the mitogen-activated protein kinase (MAPK)/extracellular signal-related kinase (ERK) [49,50,51,52,53,54,55,56,57].

Notably, both of these signaling pathways are activated by the same cytokines or growth factors that are released from the inflammatory cells infiltrating oral squamous preneoplastic and neoplastic lesions [3]. Consistently, both PI3K/AKT and MAPK/ERK result in being overactivated in OPMDs, and even more so in OSCCs, reaching levels of intensity that are directly correlated with the severity of the disease [58,59]. In particular, the greatness of AKT activation increases as OPMD progresses to OSCC, and when OSCC becomes invasive [60]. Notably, the abnormal activation of the PI3K/AKT pathway that is detected in almost all OSCCs results from the overactivation of EGF signaling or the mutation/amplification of one or more of the genes coding for the molecules involved in AKT phosphorylation [58]. Analogously to PI3K/AKT, an increased activation of ERK has been observed in non-invasive OSCC as compared to non-neoplastic oral mucosa, and in invasive OSCC as compared to non-invasive OSCC [61].

In brief terms, inflammatory cytokines or growth factors expressed in OPMDs and OSCCs turn on PI3K in epithelial cells by promoting the conversion of phosphatidylinositol 4,5-bisphosphate to phosphatidylinositol 3,4,5-trisphosphate: the latter (with the cooperation of 3-phosphoinositide-dependent protein kinase-1) phosphorylates AKT, activating it [49]. In turn, phosphorylated AKT (pAKT) turns on TWIST and, at the same time, induces the expression of SNAI both directly and indirectly, i.e., by increasing the activity of the nuclear factor–kappa B (NF-κB) transcription factor [49]. For its part, TWIST enhances the pro-EMT effects of AKT by promoting its phosphorylation [49]. Moreover, pAKT phosphorylates glycogen synthase kinase 3 (GSK-3) β, leading to its degradation and thus preventing it from deactivating SNAI [49]. A further consequence of GSK-3β degradation is the increase in the intracellular content of β-catenin, a component of cadherin-based adherents junctions, and the key effector of Wingless-related integration site (Wnt) signaling in the nucleus [49]. Under physiological conditions, β-catenin binds to the E-cadherin intracellular domain, remaining in the cytoplasm; the E-cadherin down-regulation occurring in EMT causes the nuclear translocation of β-catenin, whose transcriptional activity is then increased upon its phosphorylation via pAKT [49].

Concerning the MAPK/ERK pathway, its triggering leads to ZEB, TWIST, and SNAI expression, and it both stabilizes and phosphorylates the TWIST1 protein in epithelial cells, those lining the oral cavity included [49,50,51,52,53,54,55,56,57]. Moreover, MAPK phosphorylation switches on β-catenin signaling and, at the same time, favors NF-κB translocation into the nucleus [62,63,64]. Thus, following PI3K/AKT and MAPK/ERK activation, the nuclear levels and/or the transcriptional activity of TWIST, SNAI, NF-κB, and β-catenin increase, thus inducing EMT and promoting MMPs expression in epithelial cells, those of the oral cavity included, whether they are normal or transformed [3,65,66,67] (Figure 1). With specific regard to the oral cavity, in OPMDs, the EMT contributes to the dysplasia that characterizes these lesions [7,37,39]; in contrast, in OSCCs, the EMT is not only linked to dysplasia but also facilitates the spreading of cancerous cells first locally and then at distant sites [6].

Because of the inherent subversion of the mechanisms that control cell proliferation and the concurrent presence of growth factors in the tumor microenvironment, OSCC cells proliferate, aggrandizing the OSCC mass [3] (Figure 1).

As the tumor is enlarged, local blood vessels cannot satisfy its augmented request for oxygen and nutrients [68]. Wherefore, the tumor undergoes hypoxia, and hypoxia-inducible transcription factors (HIF)-1 and -2 are activated to stimulate angiogenesis [68] (Figure 1). Specifically, HIF-1 promotes the expression of VEGF, together with its type-2 receptor (VEGFR-2) [69,70]. An additional HIF-1 target that is transcribed in OSCC hypoxic areas is glucose transporter protein 1 (GLUT-1), a member of a family of proteins that mediate glucose’s entry into the cells [71]. Therefore, in hypoxic OSCCs, the activation of HIF-1 leads to the simultaneous expression of VEGF and GLUT-1: the former increases the number of intra-tumoral vessels, while the latter improves glucose uptake by the proliferating endothelial and OSCC cells [71]. In this regard, it must be highlighted that the energy that is required for cancer cell migration, invasion, and metastasis is predominantly provided by glycolysis [72,73]. Thus, any increase in GLUT expression would also facilitate OPMD progression to invasive OSCC, as well as the spreading of OSCC cells throughout the human body.

At variance with the *vegf* gene, those coding for FGF-2 or ANG-2 are not transcriptional targets of HIF-1 [69,74].

Concerning HIF-2, it cooperates with prostaglandin E-2 and prostacyclin-2 at stimulating endothelial cells to produce ANG-2 and invade the vascular basement membrane, thereby starting angiogenesis [74,75,76]. Because of the concurrent activation of HIF-1 and HIF-2, ANG-2 and GLUT-1 are co-expressed in colorectal cancer cells cultured under hypoxic conditions [77]. To date, it is not known whether the same phenomenon also occurs in OSCC cells.

Notably, HIF-1 can also activate the expression of the EMT transcription factors TWIST, SNAI, and ZEB [78] (Figure 1). Thus, hypoxia simultaneously promotes the trans-differentiation of epithelial cells and the angiogenesis that supports the proliferation of the trans-differentiated epithelial cells [78,79] (Figure 1).

In the oral cavity, the concurrent occurrence of these HIF-1-induced events has been shown to favor not only OSCC progression [78] but also OPMD’s evolution to OSCC [30] (Figure 1).

Of note, a reduction in HIF-1 activity is followed by the simultaneous downregulation of VEGF, SNAI, and TWIST, thus inhibiting angiogenesis and, at the same time, reverting the EMT phenotype of carcinoma cells [79]. The return of carcinoma cells to a more epithelial phenotype is termed the “mesenchymal-to-epithelial transition” (MET) [12].

In many human carcinomas, a continuous switch from EMT to MET, and vice versa, is observed [12]. Such cellular plasticity has been noted in OSCC tissues as well, and it is thought to be an adaptive response of the cancer cells to the varying tumor microenvironment [80].

It is generally believed that the EMT phenotype helps carcinoma cells invade the peritumor area, penetrate the stroma, reach and enter blood or lymphatic vessels, and survive while circulating in blood or lymph [12]. In contrast, MET occurs at the metastatic site, where it aids the metastasized cells in adapting to the novel microenvironment that they find in the colonized tissue [12]. In this context, however, it must be highlighted that, when carcinoma cells undergo MET, their invasiveness is reduced [12,44,81].

## 3. The Angiogenic VEGF, ANG-2, and FGF-2 Are Overexpressed in OPMDs and OSCCs, Where They Trigger the Molecular Pathways Leading to EMT

Previous work has reported that VEGF-A and VEGF-C expression is higher in OPMDs and OSCCs than in healthy oral mucosa [19]. Interestingly, VEGF levels increase when OPMDs evolve into OSCCs, and they rise further as OSCCs progress, positively correlating not only with the number of tumor vessels but also with the degree of cancer cell invasiveness [19,28,82]. Consistently, VEGF-A and VEGF-C expression levels in OSCC tissues are significantly associated with higher tumor stages, invasion grades, recurrence, and lymph node metastasis and, thereby, with the poor 5-year survival rate of OSCC patients [24,82].

In OSCC, VEGF is produced primarily by the carcinoma cells themselves, especially when they are subjected to hypoxia [69,70,83]. Nonetheless, it is conceivable that VEGF is also synthesized by the leukocytes that infiltrate the inflamed oral mucosa where OPMDs or OSCCs originate [3]. Indeed, T lymphocytes, neutrophils, basophils, and monocytes are all capable of producing VEGF [84,85,86]. Moreover, VEGF could also be spawned by the macrophages and keratinocytes that are activated during the inflammatory processes preceding OPMDs’ development or accompanying OPMDs’ evolution into OSCCs [3,87,88].

Like VEGF-A and -C, ANG-2 is also overexpressed in OSCCs as compared to non-cancerous peri-tumor tissue and healthy oral mucosa [28,34]. As expected, ANG-2 levels increase in OSCCs in parallel with the number of newly formed vessels [28,34]. Accordingly, ANG-2 overexpression positively correlates with the viability of OSCC cells and with the volume of the OSCC mass, while it is negatively associated with OSCC patients’ overall survival [28,34]. It is currently unknown whether OSCC cells synthesize ANG-2 as breast carcinoma cells do [88]. In this regard, one should consider that endothelial cells synthesize and release ANG-2 [89,90]. Given that, ANG-2 could be produced by the endothelial cells that line the vessels nourishing the growing OSCC.

Regarding FGF-2, its expression is induced during oral wound repair, in which FGF-2 plays a preponderant role [31,91]. Interestingly, FGF-2 and its receptors are overexpressed in OPMDs, especially in those that are at high risk of malignant transformation [29,92]. FGF-2 levels are further augmented in OSCCs [31], where the cytokine is synthesized by the tumor cells [93] and, possibly, by T cells, monocytes, macrophages, and/or inflamed keratinocytes [93,94,95,96]. Notably, FGF-2 directly sparks the survival and proliferation of OSCC cells; these pro-tumor effects add to FGF-2 angiogenic activity [97]. In contrast, either VEGF or ANG-2 promotes OSCC growth only indirectly, i.e., by stimulating angiogenesis [34,98,99].

Taken together, these findings point to VEGF, FGF-2, and ANG-2 as reliable prognostic markers for OSCC patients [19,24,28,29,31,34,82,92].

Additional data strongly support a role for VEGF, FGF-2, and ANG-2 in the EMT that characterizes OPMDs and OSCCs.

Concerning a possible role for VEGF in EMT induction, one should consider that the binding of VEGF-A to VEGFR-2 sparks a variety of intracellular signaling pathways, among which PI3K/AKT and MAPK/ERK are included [100] (Table 1).

As we mentioned before, the activation of PI3K/AKT and/or MAPK/ERK stimulates EMT [49,54,55,56,57,67]; notably, the triggering of these same signaling pathways also induces new vessel formation [100,141,142]. In this regard, a positive correlation between VEGF expression and both new vessel formation and the acquisition of the invasive EMT phenotype by oral epithelial cells has been observed in OPMDs and OSCCs [19,111].

Analogous findings have been described for other types of carcinomas where VEGF levels parallel those of SNAI, TWIST, vimentin, MMP-9, and N-cadherin and are inversely correlated with E-cadherin expression [143,144]. A similar, although less intense, phenomenon has been observed in normal epithelia in the context of inflammatory/reparative processes [145].

However, studies that have aimed to dissect VEGF’s role in EMT have produced contrasting data. Specifically, the results from several articles indicate that, rather than directly stimulating EMT, VEGF is produced by cells that have undergone EMT [18,19,36,146,147,148,149,150,151]. This finding agrees with the fact that the triggering of MAPK/ERK or PI3K/AKT leads to VEGF expression [146,147,152]. Consistently, MAPK/ERK or PI3K/AKT inhibitors can reduce VEGF expression [148,150,151,153,154]. Nevertheless, the exposure of pancreas carcinoma cells to VEGF-A is followed by the nuclear translocation of beta-catenin and an increase in SNAI and TWIST expression: this, in turn, causes pancreas carcinoma cells to acquire EMT features such as a reduction in E-cadherin levels and the concomitant induction of N-cadherin and vimentin expression [108] (Table 1). Further work is, then, needed to fully clarify whether VEGF can directly trigger EMT in normal, dysplastic, and/or neoplastic oral epithelial cells.

Regarding ANG-2, it activates ERK and AKT in carcinoma but not in normal cells [101,102,155] (Table 1). Indeed, OSCC cells that overexpress ANG-2 display reduced E-cadherin and increased vimentin levels, as well as augmented migratory/invasive capabilities [34]. Likewise, ANG-2 overexpression parallels the EMT of breast carcinoma cells in vitro, in animal models, and in oncologic patients, where it reflects the degree of differentiation, lymph node invasion, and metastasis of the tumor [101]. Analogous data have been reported for lung carcinoma cells, where silencing the ang-2 gene lowers the expression of TWIST and SNAI, thereby promoting the MET of these cancer cells [44].

Similarly to what occurs for VEGF, the expression of ANG-2 is upregulated by MAPK/ERK agonists [152] and downregulated via MAPK/ERK antagonists [153,156]. Differently from VEGF, however, the activation of PI3K/AKT downregulates ANG-2 [26], although PI3K/AKT inhibitors repress ANG-2 expression [156]. Additional investigations are required to shed light on this discrepancy.

**Figure 2:**
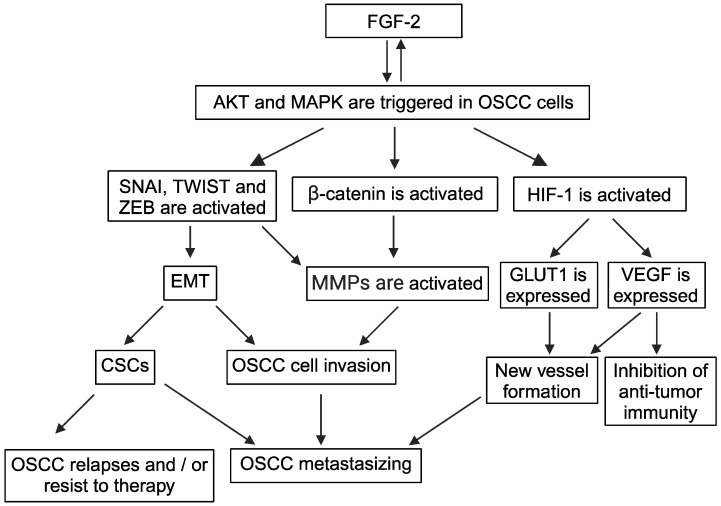
FGF-2 promotes EMT in oral mucosa. Arrows symbolize directions of connections. Abbreviations: AKT, protein kinase B; CSCs, cancer stem cells; EMT, epithelial-to-mesenchymal transition; FGF, fibroblast growth factor; GLUT, glucose transporter protein; HIF, hypoxia-inducible factor; MAPK, mitogen-activated protein kinase; MMPs, matrix metalloproteinases; OSCC, oral squamous cell carcinoma; SNAI, zinc finger snail homolog; TWIST, basic helix–loop–helix twist homolog; VEGF, vascular endothelial growth factor; ZEB, zinc finger E-box-binding homeobox. Created with BioRender.com.

At variance with VEGF and ANG-2, the role of FGF-2 in EMT, and the mechanisms for it, is clear and well established (Figure 2).

In fact, during the repair of an injured epithelium, FGF-2 already directly activates SNAI, promotes the nuclear translocation of β-catenin, lowers the levels of E-cadherin, and induces the expression of vimentin in keratinocytes, endowing them with the motility they need to close the edges of the wound [91] (Table 1). However, to fully carry out its pro-EMT action in the non-neoplastic mucosa, FGF-2 requires the help of TGFβ1 [91]. Instead, FGF-2 alone promotes EMT in OSCC cells [112] (Figure 2). Specifically, the elevated levels of FGF-2 detected in OSCCs are associated with the induction of EMT in OSCC cells [112]. This is probably because FGF-2 is overexpressed, together with its receptors in OSCCs [31,157]. In this context, it is noteworthy that the expression of FGF-2 and its co-receptors is upregulated during inflammation [158,159]. Analogously to OSCC, a direct pro-EMT effect of FGF-2 has also been observed in ovarian and liver carcinomas [109,110].

Noticeably, FGF-2 triggers either PI3K/AKT or MAPK/ERK in a wide variety of normal, as well as tumor, cells [104,105,106,107] (Table 1; Figure 2). Due to its ability to activate the PI3K/AKT signaling pathway, FGF-2 increases SNAI and ZEB expression and stabilizes TWIST by compromising its binding to GSK-3β [109,114] (Table 1; Figure 2). For its part, the activation of MAPK/ERK promoted by FGF-2 upregulates the expression of ZEB [110] (Figure 2). In this regard, one should consider that FGF-2 can directly induce the expression of vimentin and N-cadherin, while repressing that of E-cadherin [113,114] (Table 1). In agreement with these findings, FGF-2 antagonists block EMT and induce MET [160].

Interestingly, as previously mentioned for VEGF, the interactions between FGF-2 and MAPK/ERK or PI3K/AKT are also both-sided. Specifically, the activation of MAPK/ERK or PI3K/AKT is followed by FGF-2 upregulation [161,162] (Figure 2), while ERK or AKT inhibitors reduce FGF-2 expression [154,163].

Notably, at the same time at which they downregulate the angiogenic factors, the inhibitors of PI3K/AKT or MAPK/ERK signaling induce MET, decrease the expression of MMPs, and upregulate MMPs antagonists, such as the tissue inhibitor of MMPs (TIMP)-1 [150].

Regarding the hypoxia that occurs in proliferating OSCCs, one should consider that HIF-1 and VEGF can reciprocally upregulate their expression [69,74,164].

Like VEGF, FGF-2 upregulates HIF-1 expression in carcinoma cells (Figure 2), thereby potentiating HIF-1 activity and keeping it even when oxygen levels in tumor tissue are normal [165]. This effect follows the phosphorylation of AKT triggered by FGF-2 and leads to VEGF synthesis in carcinoma cells [165] (Figure 2). In addition to VEGF, FGF-2 also upregulates ANG-2 [95]: it is very likely that these activities contribute to the EMT-promoting effect of FGF-2.

In agreement with their capability of upregulating HIF-1, both VEGF and FGF-2 increase GLUT-1 expression in endothelial cells [166,167,168] (Table 1; Figure 2): as for FGF-2, it is likely that this can guarantee the optimal use of glucose via the nascent tumor vessels even in conditions of normoxia.

Moreover, given the importance of glycolysis in cancer cell locomotion [72,73], VEGF and FGF-2’s capability of upregulating GLUT-1 could facilitate OSCC metastasis.

Differently from VEGF and FGF-2, ANG-2 decreases both the expression and the DNA-binding activity of HIF-1, hence downregulating VEGF expression [169]. At present, it is not known whether ANG-2 affects GLUT-1 expression.

Taken together, the findings discussed in this section indicate that angiogenesis and EMT occurring in OSCC have strong interconnections, most of which synergize in promoting OSCC progression.

## 4. The Pro-EMT Effects of Angiogenic Factors Have a Role in OSCC Metastasizing

A few types of human cells possess motile capabilities that allow them to take part in physiological, tightly regulated reactive/reparative processes [170,171,172]. In contrast, abnormal and deregulated cell migration characterizes a variety of diseases, neoplasms included [170,171,172]. In particular, the migration through the stroma that carcinoma cells carry out after they have invaded the basement membrane is a fundamental step of the metastatic process [170].

The formation of metastases involves at first the detachment of cancerous cells from the primary tumor [173]. In OSCCs, this event is facilitated first by the decrease in E-cadherin levels on the surface of the carcinoma cells that have undergone EMT [6]. Indeed, the loss of E-cadherin loosens the adhesions among OSCC cells [6]. Another important mediator of tumor cell detachment is MMP-1, which degrades the ECM molecules that hold together the cells constituting the neoplastic mass [174]. Notably, in OSCC tissues, MMP-1 is overexpressed [175], while E-cadherin is downregulated [6].

Once detached from the primary tumor, carcinoma cells move through the peritumoral ECM via two substantially different modes, that is as single cells or as cellular aggregates [170].

When migrating as single cells, carcinoma cells generally adopt the so-termed mesenchymal mode of migration [170]. The latter strictly relies on the EMT phenotype and implies that cells rearrange their cytoskeletons via the polymerization of actin filaments and their crosslink with myosin that, in turn, strengthens the actin filaments and renders them contractile [170,171,172]. Specifically, actin polymerization drives the formation of a protrusion at the leading edge of migrating carcinoma cells [170,176,177]. Such a protrusion, which is a specialized podosome for which the newly synthesized vimentin contributes in providing a scaffold [176,177,178], is termed invadopodium because it is through it that cancer cells invade the stroma [170,176,177]. While the invadopodium firmly attaches to the ECM, the back edge of the migrating cell retracts due to myosin contraction and detaches from the ECM: this causes the cell to move forward, being attracted by growth factors and/or ECM molecules [170,171,172].

In sum, cells move via their adhesive interactions with the ECM [170,171,172]. The latter are mostly mediated by the integrins, a family of transmembrane receptors [170]. Notably, in migrating carcinoma cells, the integrins cluster on the invadopodium immediately after the latter adheres to the ECM [170,171,172]. The extracellular domain of the integrins binds to the ECM [171,172], whereas their intracellular domain interacts with the actin cytoskeleton and GTPases such as Rho, Rac, and Cdc42 [171,172,179]. At the leading edge of a migrating carcinoma cell, mechanical signals resulting from invadopodium’s adhesion to the ECM activate Rac and Cdc42, which, in turn, promote actin polymerization; at the cellular rear, the same ECM signals trigger Rho that stimulates myosin contractility [171,172].

Thanks to the joint activity of the integrins, the actin/myosin complexes, and the GTPases, carcinoma cells deform their cytoskeleton so to enhance their migratory capabilities as much as possible: then, the single carcinoma cells invade the peritumoral ECM via the proteolytic activity of MMPs and/or uPA [3,10,15,16,173].

Amidst MMPs, the MMP-2, MMP-9, and membrane type (MT) 1-MMP are expressed in OSCC at levels that positively correlate with the size, histologic grade, or stage of progression of OSCC and are predictive of OSCC metastasizing [3]. While MMPs are overexpressed, their antagonists TIMPs are downregulated in OSCCs as compared to the healthy oral mucosa [180]: definitely, these two concurrent phenomena strongly increase the activity of MMPs in the tumor tissue.

Analogously to the MMPs, uPA and its receptor (uPAR) are also expressed in OSCCs at levels that positively correlate with the invasive and metastatic capabilities of these tumors [15].

Notably, the ECM-degrading MT1-MMP or uPA and the ECM-binding integrin receptors are positioned in proximity on the invadopodium of OSCC cells: this drives ECM degradation toward the direction that the cancer cells are heading [3]. Noteworthy, the invadopodium is found not only on the surface of OSCC cells but also on that of the keratinocytes constituting OPMDs at high risk for malignant transformation [181].

Due to the activity of MMPs and/or uPA, carcinoma cells create real tunnels in the ECM [170]. Migrating single carcinoma cells that have undergone EMT can pass through those tunnels according to the so-called amoeboid migration: the latter, typical of leukocytes, is very fast and almost independent of cellular adhesions [170]. Therefore, individually migrating carcinoma cells can change their migration mode from mesenchymal to amoeboid, depending on the state of ECM integrity [170].

In addition, the paths generated by MMPs and/or uPA allow the migration of carcinoma cells grouped in sheets or filaments that would otherwise be stopped due to the physical barrier constituted by an intact ECM [170].

In the collectively migrating cancer cells, a front is distinguished that consists of cells with an EMT phenotype that tow cells with a fairly epithelial phenotype, which are connected to each other via epithelial intercellular adhesion molecules such as E-cadherin [170]. Interestingly, invadopodia are located on the ventral surface of collectively invading cancer cells, and they release a large number of MMPs to favor collective invasion [178].

It must be highlighted that carcinomas metastasize via collective migration more than via single-cell migration and that carcinoma cells with an epithelial phenotype are very viable and aggressive, although they are less mobile than those that have undergone EMT [81,170].

Concerning OSCCs, results from ex vivo and in vitro studies indicate that both single-cell and collective migration occurs in this tumor and that the mode of migration is strongly influenced by the EMT phenotype of the cancer cells [47,81,182]. Specifically, vimentin-positive OSCC cells with low levels of E-cadherin are very invasive and adopt the single-cell migration mode; in contrast, vimentin-negative OSCC cells expressing high levels of E-cadherin are minimally invasive and migrate collectively [47,81,182].

Regarding a possible direct effect of angiogenic factors on the locomotion of normal and/or transformed oral epithelial cells, VEGF stimulates the migration of OSCC cells but not of normal oral keratinocytes [183], while FGF-2 appears to be involved in the motility of both normal and neoplastic oral epithelial cells [140,184]. As for ANG-2, we have found no information on whether it has a direct chemotactic effect on OSCC cells or oral keratinocytes. Nonetheless, given EMT’s role in carcinoma cell migration, and considering the links that VEGF, FGF-2, and ANG-2 have with EMT, all three angiogenic factors could likely contribute to OSCC metastasizing. This hypothesis is supported by the finding that VEGF, FGF-2, or ANG-2 expression markedly increases in OSCCs immediately before and during the metastatic process [19,31,34,82].

In this context, one should consider that VEGF, FGF-2, or ANG-2 are all capable of inducing or upregulating MMP-1 expression [117,118,119], as well as repressing E-cadherin [19,34,111,112] (Table 1; Figure 2). Thus, angiogenic factors may favor the detachment of OSCC cells from the primary tumor by both reducing E-cadherin levels and upregulating MMP-1.

Still in this regard, previous work has reported that VEGF and FGF-2, but not ANG-2, trigger Rho GTPases to promote cellular migration [185,186]. Consistently, VEGF activates actin filament polymerization, vimentin expression, and invadopodium formation at the migrating front of cancer cells in the same way in which it promotes the budding of a podosome from the surface of endothelial cells during angiogenesis [108,187,188,189,190]. Analogous findings have been described for neuronal cells upon their exposure to FGF-2 [191]. ANG-2 could also play a role in this important step of the metastatic process, given that the production and release of ANG2 are followed by the formation of podosomes in endothelial cells [192]. In addition, VEGF or ANG-2 upregulates (while FGF-2 inhibits) myosin expression in stem cells [193,194,195]. Moreover, VEGF and ANG-2 are, respectively, involved in the cellular locomotion mediated by beta 1 and beta 3 integrins [196,197], which are the same clustering on the invadopodium [170].

Further investigations are needed to clarify whether VEGF, FGF-2, and/or ANG-2 promote all these events in OSCC cells.

For now, it is well established that VEGF, FGF-2, and ANG-2 upregulate MT1-MMP, MMP-2, and MMP-9 expression (Table 1; Figure 2): consistently, a positive correlation has been observed between the levels of these angiogenic factors and those of MT1-MMP, MMP-2 or MMP-9 in tumor tissues [120,121,122,123,124,125,126,127,128,129,130,131,132]. In addition, VEGF, FGF-2, and ANG-2 are known to reduce TIMP-1 levels in vitro and in vivo [133,134] (Table 1; Figure 2).

Notably, VEGF levels mirror those of uPA in neoplasms [198], especially where NF-κB is overactivated [199]. Regarding FGF-2, it can directly upregulate uPA expression [200], while ANG-2 levels positively correlate with those of uPA and uPAR in tumor specimens, as well as during wound healing [201,202].

Additional work must be performed to assess whether VEGF, FGF-2, and ANG-2 diminish the levels of TIMPs and increase those of uPA/uPAR in dysplastic or neoplastic oral epithelial cells.

While OSCC cells degrade the peritumoral ECM and move through it, the ECM changes in its components. Specifically, laminin (LN)-5 is deposited on the basement membrane [203], and the content of tenascin (TN), fibronectin (FN), and WNT1-inducible signaling pathway protein-1 (WISP-1) in the ECM increases [17,36,203,204]. In this context, LN-5, TN, and FN are synthesized by OSCC cells and/or OSCC-associated fibroblasts with the cooperation of inflammatory cells [203,204,205,206]. As for WISP-1, it must be highlighted that it is produced upon the activation of the Wnt-1/beta-catenin pathway [207], which is constitutively triggered in progressing OSCC [3,67]. Interestingly, WISP-1 activates AKT and SNAIL, thereby stimulating the EMT of oral keratinocytes [17]. In addition, WISP-1 promotes both lymphangiogenesis and the migration of OSCC cells, thereby favoring OSCC metastasizing [17]. For its part, LN-5 promotes the locomotion of OSCC cells toward the basement membrane [203], while TN provides OSCC cells that have acquired the motile EMT phenotype with a solid support that facilitates their migration [204]. As for FN, it is present mostly at the invasive front of OSCCs, where it increases the speed of carcinoma single-cell migration [81]. Moreover, FN binding to its main receptor, which is the α₅β₁ integrin, triggers the expression of the anti-apoptotic BCL-2 protein by carcinoma cells, thereby favoring their survival [208].

Among all the cytokines present in the OSCC microenvironment, TGFβ1 is particularly effective in inducing the changes in the peritumoral ECM that accompany the metastasis process [3,209].

Concerning the possibility that angiogenic factors could contribute to the changes in ECM composition observed in OSCC tissues, it is noteworthy that FGF-2 upregulates both FN and α₅β₁ [114,116] (Table 1). In contrast, VEGF does not induce FN expression (Table 1); it is, rather, FN, as well as WISP-1, that promotes VEGF synthesis by OSCC cells [36,115].

Regarding ANG-2, its overexpression in human gliomas parallels an increase in LN-5, and this correlates with the upregulation of MT1-MMP and MMP-2, as well as the invasion of glioma cells [210]. It is currently unknown whether these events also occur in OSCC.

The fact that VEGF, FGF-2, and/or ANG-2 induce the EMT phenotype leads us to imagine them as involved mainly in single-OSCC cell migration. This hypothesis is supported by the links existing between VEGF or FGF-2 and the Yes-associated protein 1 (YAP), a component of the Hippo pathway that is inactivated during the collective migration of OSCC cells [211]. In particular, FGF-2 activates YAP [212], which, in turn, inhibits VEGF expression [213].

However, one should consider that EMT cells are present at the front of migrating carcinoma cell aggregates [170]. In this regard, it must be highlighted that hypoxia promotes the expression of VEGF, together with that of discoidin domain receptor-1, a membrane receptor that orchestrates the collective migration of carcinoma cells and that is highly expressed in OSCCs [214,215,216]. Furthermore, VEGF can bind the alpha v integrins [217], which mediate the aggregation and collective migration of OSCC cells [214]. Future studies could dissect the role that angiogenic factors play in each of the migration modes of OSCC cells.

After invading the stroma, OSCC cells move toward the blood or lymphatic vessels, adhere to their wall, penetrate it due to the proteolytic activity of uPA and the MMPs, and eventually disseminate throughout the organism via the blood or lymph [155]. There, carcinoma cells circulate individually or in aggregates: the latter are more aggressive than single cells, and their presence in the blood or lymph is associated with the patient’s poor prognosis [170]. In this context, the alpha v integrins are believed to play a role in the intra-vascular spreading of OSCC cell aggregates [214]: this leads us to hypothesize that VEGF’s capability of binding the alpha v integrins may impact on this mode of metastasis [217].

However, neither blood nor lymph provides OSCC cells with the survival signal resulting from AKT and/or ERK activation that follows cellular adhesion to a solid ECM [218]. The lack of this signal makes circulating OSCC cells undergo “anoikis”, a peculiar type of apoptosis that can jeopardize cancer metastasizing [173,218].

When thinking about a way in which angiogenic factors could help OSCC cells overcome anoikis, one should consider that VEGF levels are high in the serum of OSCC patients [219,220] and that OSCC cells express VEGFR-2 [221]. Indeed, VEGF binding to VEGFR-2 activates AKT-mediated cell survival signals [221]: this would increase the viability of circulating OSCC cells, protecting them from anoikis, as has been reported for other carcinoma cell types [135,136] (Table 1). Therefore, VEGF could facilitate OSCC metastasizing both by increasing the viability of circulating cancer cells and by promoting the formation of new vessels that function as metastasis routes.

As for ANG-2, it is present in the serum of OPMD patients [222]. Given its capability of triggering survival signals in transformed cells [119], ANG-2 would also be of importance for OSCC metastasizing. However, we have found no information on whether ANG-2 is present in the serum of OSCC patients.

FGF-2 has been reported to counter the anoikis of human stem cells because of its ability to ignite either AKT or ERK [137] (Table 1). However, FGF-2 levels increase in the saliva, but not in the serum, of OSCC patients, as compared to healthy individuals [223]. This finding is consistent with the fact that, after its production by tumor cells and tumor-infiltrating leukocytes, the majority of FGF-2 remains within OSCC [93,224], probably because of its high affinity for the heparan sulfate proteoglycans of the cell surface and ECM [225]. Therefore, FGF-2 is likely to be less involved than VEGF in protecting circulating OSCC cells from anoikis.

## 5. Angiogenic Factors Could Favor the Establishment of Metastases at Ectopic Sites

At the end of the metastatic process, OSCC cells arrive in body areas that may be close to, or distant from, the primary tumor: there, the OSCC cells exit the vessel due to uPA and MMPs actions, adhere to local ECM, and then proliferate to give rise to secondary tumors [173]. However, for the metastasis to successfully settle in the new site, OSCC cells must defeat the attack of resident immune cells.

In this context, VEGF exerts multiple activities that help metastatic tumor cells to survive the antitumor immune responses. Specifically, VEGF impairs the maturation of T cells and dendritic cells and stimulates tumor-associated macrophages to release immunosuppressive cytokines [226]. Moreover, when combined with ANG-2, VEGF lowers NK cell activity [227]. For its part, FGF-2 could contribute to these immunosuppressive effects because of its capability to promote VEGF expression [228] (Figure 2). Altogether, these activities are likely to take place in OSCCs, where VEGF, FGF-2, and ANG-2 are concomitantly overexpressed.

The final step of the metastatic process implies that cancer cells adapt to the new environment they have arrived in, which is often very different from the one where they originated [173,229].

In this regard, the persistence of intense pro-EMT stimuli over time causes carcinoma cells to lose most of their epithelial markers and to express in their place stem cell markers, which add to the EMT ones [112]. In view of their poor differentiation, such cells are named cancer stem cells (CSCs) [229]. In other instances, normal stem cells are recruited from the bone marrow into the stroma of tumors [230], where they may eventually undergo malignant transformation, hence becoming CSCs [231]. The bone marrow-derived stem cells that infiltrate tumors produce ANG-2 [230] which, in turn, promotes angiogenesis, EMT, and tumor cell invasion and growth [26,27,34,44,232]. Notably, angiogenic factors are important to the generation and maintenance of CSCs. Specifically, due to its capability of directly inducing EMT, FGF-2 increases the number of CSCs in a population of SCC cells and, together with VEGF, strengthens their viability and self-renewal [138,139] (Table 1; Figure 2).

Like normal stem cells, CSCs have a highly plastic phenotype, which allows them to adapt even to environments that would otherwise be “hostile” [173,229]. In addition to being very mobile and invasive, CSCs have an almost infinite capacity for self-renewal [229]. Moreover, they are very viable, making them extremely resistant to anoikis [229]. Taken together, these features render CSCs particularly capable of metastasizing.

OSCC is populated by CSCs that display EMT features (such as upregulated TWIST1, newly synthesized N-cadherin, and downregulated E-cadherin), together with stem cell markers (e.g., OCT4 and SOX2) and cell surface molecules (e.g., CD44 and CD133) that are also expressed by CSCs from other types of carcinomas [233]. The CSCs of OSCCs synthesize VEGF, FGF-2, and ANG-2 [233]. FGF-2 has an undebatable role in maintaining the stemness, self-renewal capacity, and tumorigenicity of CSCs in OSCC [140] (Figure 2). Given that, and in view of what happens in other carcinomas, VEGF and ANG-2 could also positively act on OSCC-resident CSCs to promote OSCC progression.

Consistent with CSCs’ capability of surviving apoptosis, self-renewing, invading, and migrating, the presence of CSCs in an OSCC correlates with the likelihood that the tumor will recur after surgical removal and metastasize [234]. Moreover, the great viability of CSCs makes them resistant to anti-tumor radiotherapy and chemotherapy [235]. In this regard, one should also consider that EMT transcriptional activators such as TWIST, SNAI, and ZEB induce the expression of anti-apoptosis molecules, as well as genes involved in the multidrug resistance of tumor cells [49].

For all these reasons, CSCs are one of the major obstacles to the successful treatment of tumors, OSCC included [49,234,235]. Thus, because of their positive interconnections with the EMT process, FGF-2, VEGF, and/or ANG-2 could compromise the efficacy of conventional anti-OSCC therapies.

## 6. Conclusions and Future Directions

Analogously to what happens for other tumor types, the early detection of OSCC and the timeliness of the start of therapy favor the patient’s survival [236]. OSCC treatment involves the surgical excision of the tumor and, possibly, radiotherapy and/or the administration of combined chemotherapeutics, such as cisplatin, fluorouracil, and antibodies directed against EGF receptors or the programmed death 1 immune checkpoint (PD-1) protein [1,58]. However, in patients with OSCC in an advanced stage of progression, surgery is very mutilating and often incapable of preventing tumor recurrence or metastasis [1]. Furthermore, most of the late-stage OSCC patients develop drug- or radio-resistance over time [1,237]. As a result, nowadays, the five-year survival of patients with advanced OSCC has remained the same as it was sixty years ago, i.e., it does not exceed fifty percent [238]. Therefore, the identification and exploitation of novel anti-OSCC therapeutic tools, hopefully more effective than the current ones, are urgent.

In this context, curative approaches countering the two major cornerstones upon which the clinical progression of OSCC is based, namely the EMT of carcinoma cells and the neoformation of tumor-associated lymphatic and blood vessels [6,7,31], should be designed and evaluated.

Prompted by this need, here, we have discussed published findings indicating that VEGF, FGF-2, and ANG-2 are upregulated in OPMD and OSCC, where they activate the PI3K/AKT and MAPK/ERK pathways that, in turn, lead to EMT and concomitant vessels generation.

Given that PI3K/AKT and MAPK/ERK are dysregulated in a large variety of human tumors, several inhibitors of these pathways have been formulated to date, some synthetic and some from natural sources [239,240].

As for OSCC, results from experimental work indicate that ERK, p38MAPK, or PI3K/AKT inhibitors are all capable of reducing or halting the growth and migration of OSCC cells [241,242,243,244]. Moreover, these inhibitors enhance the sensitivity of OSCC cells to anticancer drugs [242,245,246].

However, it must be highlighted that resistance often develops against MAPK inhibitors, thus limiting their therapeutic efficacy [240]. Regarding this last aspect, PI3K/AKT inhibitors have proven to sensitize OSCC cells to anticancer chemotherapeutics even when these tumor cells display activated MAPKs [246].

This gives hope that PI3K/AKT inhibitors can be used in the therapy of OSCC, especially considering the importance that this signaling pathway plays in the development and progression of OSCC [58]. Certainly, however, PI3K/AKT inhibitors cannot be administered as a monotherapy, given that the intrinsic characteristics of OSCC and those of its microenvironment cause the continuous reactivation of AKT [58].

In this regard, the results of many studies support the administration of PI3K/AKT inhibitors (e.g., flavanones) in combination with conventional cytostatic/cytotoxic chemotherapeutics for the treatment of OSCC patients [58,239].

In view of the findings summarized in the present review, which point to angiogenic factors as targets for innovative therapies directed against OSCC, OSCC patients could take PI3K/AKT inhibitors also in combination with anti-angiogenic drugs [120,247]. In this regard, a phase I clinical trial carried out on twenty patients with locally advanced resectable OSCC treated with three cycles of APATINIB (a VEGFR2 inhibitor) combined with CAMRELIZUMAB (an anti-PD-1 antibody) has given promising results [248]. Specifically, all patients tolerated this treatment well, and 40% of them showed a minimal percentage of residual viable OSCC cells. Furthermore, two years after starting treatment, 95% of responder patients were still alive [248]. These clinical findings are consistent with the results of previous work highlighting the efficacy of VEGF antagonists in preclinical models of OSCC [249,250].

Alternatively, or additionally, multidrug combinatorial therapies could also include the use of steroidal and non-steroidal anti-inflammatory drugs that have been shown to counter AKT [204] and promote MET [79,251].

Since the MET phenotype generally results in a decrease in the invasive capabilities of carcinoma cells [49,252], it would be a great success to find a therapy reverting the EMT of the highly metastatic OSCC cells to a more static phenotype. Indeed, EMT not only facilitates cancer cell invasion and dissemination throughout the body but also augments cancer cells’ resistance to chemotherapy or radiotherapy [49,253,254]. Therefore, it is believed that converting the OSCC cell phenotype to a more epithelial-like one would not only reduce OSCC invasiveness and metastasizing but also render OSCC cells more sensitive to anticancer therapies [252]. This would be particularly beneficial, given that OSCC patients frequently develop radio- and/or chemo-resistance [1,237].

Finally, considering the easy accessibility of the oral cavity, anti-EMT and anti-angiogenic drugs may find a topical application that could possibly reduce their side effects.

## Figures and Tables

**Table 1:** VEGF-A, ANG-2, or FGF-2 exerts EMT-associated, pro-tumor activities.

DIRECT EFFECT	VEGF-A	ANG-2	FGF-2
Triggering of AKT or MAPK	Yes [100]	Yes [101,102,103]	Yes [104,105,106,107]
Activation of TWIST, SNAI, or ZEB	Yes [108]	Not known	Yes [91,109,110]
Downregulation of E-cadherin expression	Yes [19,108,111]	Yes [34]	Yes [112]
Induction of N-cadherin expression	Yes [108]	Not known	Yes [113]
Induction of vimentin expression	Yes [108]	Yes [44]	Yes [114]
Induction of fibronectin expression	No [36,115]	Not known	Yes [113,116]
Upregulation of MMP-1	Yes [117]	Yes [118]	Yes [119]
Upregulation of MT1-MMP, MMP-2, and MMP-9	Yes [120,121,122,123,124,125,126,127,128]	Yes [129]	Yes [128,130,131,132]
Downregulation of TIMP-1	Yes [133]	Yes [133]	Yes [134]
Protection from anoikis	Yes [135,136]	Not known	Yes [137]
Promotion of CSCs appearance and viability	Yes [138,139]	Not known	Yes [138,139,140]
